# Psychotic (delusional) depression and completed suicide: a systematic review and meta-analysis

**DOI:** 10.1186/s12991-018-0207-1

**Published:** 2018-09-21

**Authors:** Rossetos Gournellis, Kalliopi Tournikioti, Giota Touloumi, Christos Thomadakis, Panayiota G. Michalopoulou, Ioannis Michopoulos, Christos Christodoulou, Athanasia Papadopoulou, Athanasios Douzenis

**Affiliations:** 1 Second Department of Psychiatry, Medical School, National and Kapodistrian University of Athens, University General Hospital “ATTIKON”, 1 Rimini Str TK 12462, Athens, Greece; 2 Department of Hygiene, Epidemiology and Medical Statistics, Medical School, National and Kapodistrian University of Athens, Athens, Greece; 3 Cognition, Schizophrenia, Imaging Laboratory, Department of Psychosis Studies, Institute of Psychiatry, Psychology and Neuroscience King’s College London, London, UK

**Keywords:** Psychotic depression, Unipolar depression, Suicide, Systematic review, Meta-analysis

## Abstract

**Background:**

It remains unclear whether psychotic features increase the risk of completed suicides in unipolar depression. The present systematic review coupled with a meta-analysis attempts to elucidate whether unipolar psychotic major depression (PMD) compared to non-PMD presents higher rates of suicides.

**Methods:**

A systematic search was conducted in Scopus, PubMed, and “gray literature” for all studies providing data on completed suicides in PMD compared to non-PMD, and the findings were then subjected to meta-analysis. All articles were independently extracted by two authors using predefined data fields.

**Results:**

Nine studies with 33,873 patients, among them 828 suicides, met our inclusion criteria. PMD compared to non-PMD presented a higher lifetime risk of completed suicides with fixed-effect pooled OR 1.21 (95% CI 1.04–1.40). In a sub-analysis excluding a very large study (weight = 86.62%), and comparing 681 PMD to 2106 non-PMD patients, an even higher pooled OR was found [fixed-effect OR 1.69 (95% CI 1.16–2.45)]. Our meta-analysis may provide evidence that the presence of psychosis increases the risk of suicide in patients suffering from severe depression. The data are inconclusive on the contribution of age, mood congruence, comorbidity, and suicide method on PMD’s suicide risk. The lack of accurate diagnosis at the time of suicide, PMD’s diagnostic instability, and the use of ICD-10 criteria constitute the main study limitations.

**Conclusions:**

The presence of psychosis in major depression should alert clinicians for the increased risk of completed suicide. Thus, the implementation of an effective treatment both for psychotic depression and patients’ suicidality constitutes a supreme priority.

**Electronic supplementary material:**

The online version of this article (10.1186/s12991-018-0207-1) contains supplementary material, which is available to authorized users.

## Background

Being affected by a mental disorder is a significant risk factor for suicide. Therefore, the identification of patients at risk for suicide among patients with mental disorders is of high clinical relevance. Psychological autopsy studies have shown that depression is the most common mental disorder at the time of suicide, occurring in half to two-thirds of cases [1–4]. Unipolar depression has been associated with increased risk for suicide. In fact, suicide accounts for nearly 20% of the deaths in psychiatric samples of depressed patients [5]. From a clinical point of view, the severity of depressive symptoms has been positively related to suicide [6].

The presence of psychotic features (delusional ideas and/or hallucinations) during a major depressive episode defines psychotic major depression (PMD) [7]. Unipolar PMD compared to non-PMD is a more severe form of depression with greater feelings of guilt and more pronounced psychomotor disturbance. In addition, PMD compared to non-PMD patients were found to be more severely cognitively impaired with greater HPA axis abnormalities and with lower serum dopamine *β*-hydroxylase activity. Besides, PMD is associated with more brain atrophy and poorer short-term outcome with respect to daily functioning, residual depressive symptoms, mortality, and suicidality (see [8–10] for reviews).

As regards completed suicide (henceforth suicide), studies have reported conflicting results: some comparing suicides between PMD and non-PMD patients have shown that psychosis increases the risk of suicide [11–13], whereas other studies failed to show such an effect [14–19]. However, no study has reported decreased suicide risk in unipolar PMD patients.

A recent systematic review of our group coupled with meta-analysis has shown that the presence of psychosis during a major depressive episode doubles the risk of a suicide attempt both lifetime and in the acute phase of the disorder [20]. In the same direction, Zalpuri and Rothchild [21] in a recent systematic review reached the same conclusion. Taking into consideration that a history of suicidal attempt increases the risk of a future suicide at 30–40 times compared with the risk in general population [22, 23], these findings stress further the need to investigate and quantify the impact of psychosis on suicides in unipolar depressed patients.

Thus, the aim of the current study was to elucidate further through a systematic review coupled with meta-analysis whether the lifetime risk for suicide of unipolar PMD compared to non-PMD patients is increased. An additional purpose of this study was to investigate whether the suicide risk in PMD in comparison to non-PMD patients is associated with (a) age, (b) depression severity, (c) mood congruence of psychotic features, (d) use of more violent methods, and (e) the existence of comorbidity either psychiatric and/or physical.

## Methods

We performed a systematic review of the literature in Scopus and PubMed using various combinations of the key words “psychotic depression”, “depression with psychotic features”, “delusional depression”, and “suicide” (see Appendix-Additional file 1). In addition, databases of the so-called “gray literature” such as CINAHL Complete, ProQuest Dissertations @ Theses Database, Open Gray, ERIC, NIOSH, ClinicalTrials.gov, Agency for Healthcare Research and Quality, National Institutes of Health, and Google advanced were searched. Reference lists from all relevant studies were also hand searched for additional relevant articles. This search was run until 03th August 2018. No limitation in the search strategy was inserted. We developed a data extraction sheet and refined it accordingly. Titles and abstracts of the articles were screened, and the full texts of the selected articles were reviewed and assessed by two researchers independently (RG and KT) in relevance to our inclusion and exclusion criteria. Information was extracted from each study and included characteristics of participants (number, age), phase of the disorder, length of follow-up, severity of depressive episode, diagnostic criteria, method of suicide used, mood congruence of psychotic features, and comorbidity. In cases of doubt, articles were discussed in a consensus meeting (RG, KT, and PM).

Only studies in English language providing information on suicides of unipolar PMD and non-PMD patients were included. Studies on suicidal ideation and attempts, case reports, studies that did not present primary data (for example comments, letters and reviews), and studies whose population consisted of participants with schizophrenia, delusional disorder, bipolar disorder, schizoaffective disorder, anxiety disorders, or dementia were excluded. We also excluded articles which reported data on overlapping cohorts, and we included the most representative and informative articles of the same and/or overlapping samples. Participants had to have been diagnosed with unipolar major depression according to the RDC, ICD-9, and DSM-III and beyond. The study followed the PRISMA guidelines [24], with the exception of not having registered its protocol in a Web address in advance.

### Statistical analyses

The primary outcome was defined by the odds ratio (OR) for a completed suicide comparing PMD with non-PMD patients. To generate the pooled OR, we initially used fixed-effect meta-analysis followed by random-effect models in cases of significant heterogeneity across studies [25]. To visualize individual ORs, we used forest plots, whereas, to test for small-study effects, we employed funnel plots along with Egger’s test. Statistical heterogeneity across studies was formally assessed using the corresponding Chi-square test [26], whereas the *I*^2^ statistic was used to quantify heterogeneity. All analyses were performed using the commands metan, metabias, metafunnel, and metareg in STATA (Version 13.0, Stata Corp, College Station, Texas).

## Results

The search yielded 2242 in Scopus, 1646 in PubMed, and 746 items in the “gray literature” (Google: 260, edu: 162, org: 259, gov: 75). Forty-seven studies were left for further investigation after exclusion of duplicates and irrelevant to the research question references. Twenty-five reported data on attempted suicides either on samples with unipolar [15, 18, 27–40] or unipolar and bipolar patients [41–49], while ten assessed suicidality by means of psychometric scales with no categorical data or without comparison group [50–59].

**Fig. 1:**
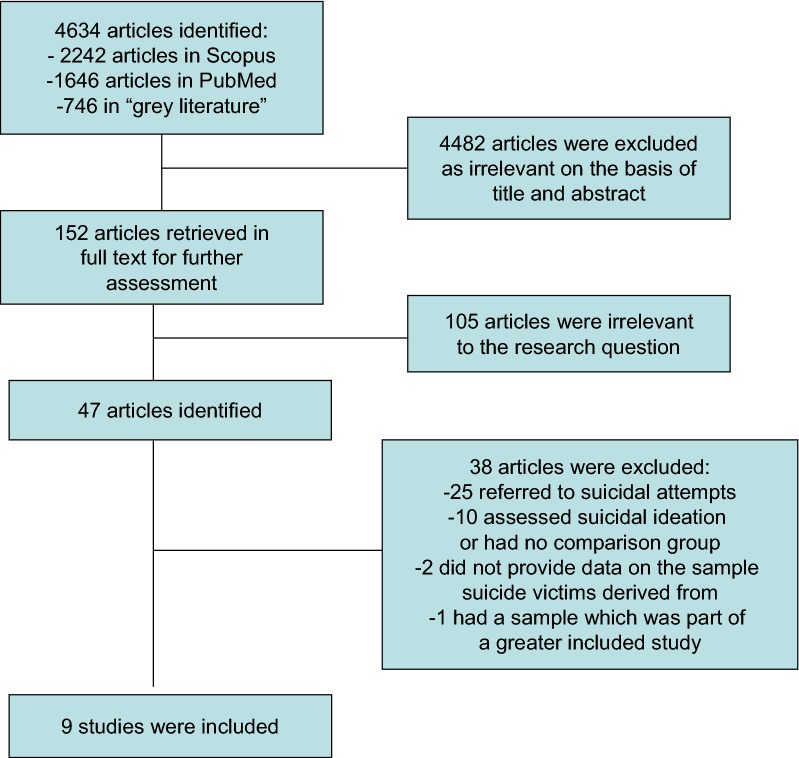
Flowchart of meta-analysis study selection

From the remaining 12 studies which reported data on completed suicides, one was excluded [60], because its sample was part of another greater included study [17] and two [13, 61], because they reported numbers of suicide victims but without providing data on the sample from which these numbers were derived from Fig. 1.

### Results from the meta-analytic leg of the study

#### The pooled-effect OR across all studies

Thus, nine studies [11, 12, 14, 16, 17, 19, 62–64] fulfilled our inclusion criteria (Table 1) and were subjected to the meta-analysis. The first meta-analysis concerns the general comparison between PMD and non-PMD patients regardless of depression severity with patients’ numbers as they were reported by the authors: the estimated fixed-effect pooled OR of these nine studies was 1.17 (95% CI 1.03–1.34, fixed-effects *p* = 0.020) with the corresponding random-effect pooled OR being 1.66 (95% CI 1.00–2.76, random-effects *p* = 0.049). However, the heterogeneity across studies was of significant size (*I*^2^ statistic = 63%, *p* = 0.006). In an effort to explain the high level of heterogeneity, we observed that, in the Leadholm et al.’s [17] study, the PMD patients (all classified as suffering from severe depression according to ICD-10 criteria) were compared to non-PMD patients manifesting also severe depression. In the Black et al. [14] and Coryell and Tsuang [16] studies, the authors do not provide data on depression severity, but the clinical characteristics of the included patients imply inpatients manifesting severe depression, as well. Schneider et al. [64] compared three suicide victims PMD patients out of 60 PMD patients to 13 suicide victims out of 218 non-PMD patients. The authors do not display specific numbers regarding depression severity; nevertheless, the majority (148) of these 278 in total patients suffered from melancholic subtype of depression, a clinical concept very close to severe depression. In contrast, in the Suominen et al.’s [12] study, 19 victims out of 110 PMD patients were compared to 53 victims out of 912 non-PMD patients suffering from mild to severe depression. From the 912 non-PMD patients, only the 447 suffered from severe depression. In all the rest studies, the authors do not provide data on depression severity and the patients’ numbers are too small and inconclusive [19, 62, 63].

Thus, to reduce heterogeneity, in a second meta-analysis, we included the Suominen et al. [12] study’s patients suffering only from severe depression (110 PMD vs. 447 non-PMD). Then, the pooled fixed-effects becomes 1.16 (95% CI 1.01–1.32), which still implies a significant difference between PMD and non-PMD patients (*p* = 0.036). The heterogeneity across studies becomes moderate (*I*^2^ = 38.7%) and non-significant (*p* = 0.110), and the random-effect pooled OR becomes 1.45 (95% CI 0.99–2.14), being marginally non-statistically (*p* = 0.057). Thus, in the subsequent analyses, we used the Suominen et al.’s [12] comparison among patients with severe depression.

However, an additional methodological issue arises: in the Leadholm et al.’s [17] study, PMD and non-PMD episodes were evaluated over the course of the disorder and, in their statistical analysis, some patients who experienced both PMD and non-PMD episodes contributed to both groups. In the subsequent analyses, we excluded the patients who had both diagnoses based on the following steps: (a) definition of denominators: in the Leadholm et al.’s [17] study, it is clarified that the total sample consisted of 34,671 patients, whereas there were 12,150 PMD and 26,106 non-PMD patients resulting in a total of 38,256 patients; thus, 3585 patients received both diagnoses. We estimated that the total number of patients having only one or more PMD episode(s) was 8565 (PMD patients: 12,150 − 3585 = 8565) and the total number of patients with only one or more non-PMD episode(s) was 22, 521 (non-PMD: 26,106 − 3585 = 22,521), (b) definition of numerators: in total, 755 patients committed suicide. In the Zalpuri and Rothschild [21] review, it was reported that, among the 12,150 PMD and the 26,106 non-PMD patients, there were 280 and 551 completed suicides, respectively. Thus, 76 patients who committed suicide had both diagnoses (831 − 755 = 76). As in the step a, we estimated that there were 204 completed suicides among the 8564 PMD patients (PMD patients who committed suicide 280 − 76 = 204) and 475 suicidal patients (551 − 76 = 475) among the 22,521 patients who had only non-PMD diagnosis. Consequently, these numbers were included in our main analysis.

**Fig. 2:**
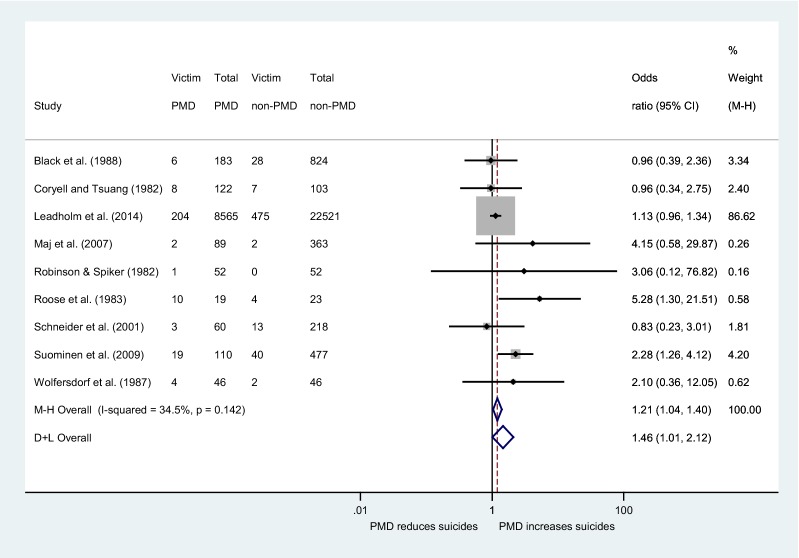
Forest plot of study-specific odds ratios for completed suicide. The term ‘M–H’ stands for the fixed-effect approach using the Mantel–Haenszel method, whereas the term ‘D + L’ stands for the random-effect approach

The estimated fixed-effect pooled OR of these nine [11, 12, 14, 16, 17, 19, 62–64] studies became 1.21 (95% CI 1.04–1.40), indicating statistically significant difference between PMD and non-PMD patients (*p* = 0.015). The heterogeneity across studies was of medium size (*I*^2^ statistic = 34.5%) albeit not statistically significant (*p* = 0.142) with the corresponding random-effect pooled OR being 1.46 (95% CI 1.01–2.12). Taking into account the *p* value of the Egger’s test (*p *= 0.141), along with the funnel plot, we concluded that there is no significant small-study effect. Consequently, this analysis constitutes the main analysis of our study (Fig. 2).

Furthermore, the weight of studies in our main meta-analysis which report that they compare patients suffering only from severe depression [12, 17] is 90.82%. In addition, the Suominen et al. [12] study is the only one that reports separately the numbers of suicide victims in each depression subtype. By comparing the 59 suicide victims out of 587 patients all suffering from severe depression (PMD and non-PMD with severe depression) to 13 suicide victims out of 435 patients suffering from non-PMD of medium and mild severity of the Suominen et al.’s [12] study, the OR was 3.63 (1.96–6.70). Then, we compared the 19 PMD suicide victims out of 110 PMD patients to the 53 non-PMD suicide victims out of 912 non-PMD patients of all degrees of severity, and we found an OR 3.38 (1.92–5.97). Finally, in a comparison of 19 PMD victims of suicide out of 110 PMD patients to 40 suicide victims out of 447 non-PMD patients presenting severe depression, the OR was 2.28 (1.26–4.12).

Besides, we carried out an additional meta-analysis with the group of PMD patients of the Leadholm et al.’s [17] study consisted of 12,150 patients, 8565 with only PMD episode(s) and 3585 patients presenting both PMD and non-PMD episode(s). The group of non-PMD is consisted of 22, 521 patients with only non-PMD episode(s) (26,106 − 3585 = 22,521). Thus, the PMD group comprises patients who presented at least once a PMD episode in the course of the disorder and the non-PMD group only non-psychotic episodes. The estimated fixed-effects pooled OR became 1.16 (95% CI 1.01–1.33), indicating statistically significant difference between PMD and non-PMD patients (*p* = 0.037). The heterogeneity across studies was of medium size (*I*^2^ statistic = 38.5%) albeit not statistically significant (*p* = 0.112) with the corresponding random-effect pooled OR being marginally not significant 1.45 (95% CI 0.99–2.14) (see Additional file 1).

#### Sensitivity analysis

**Fig. 3:**
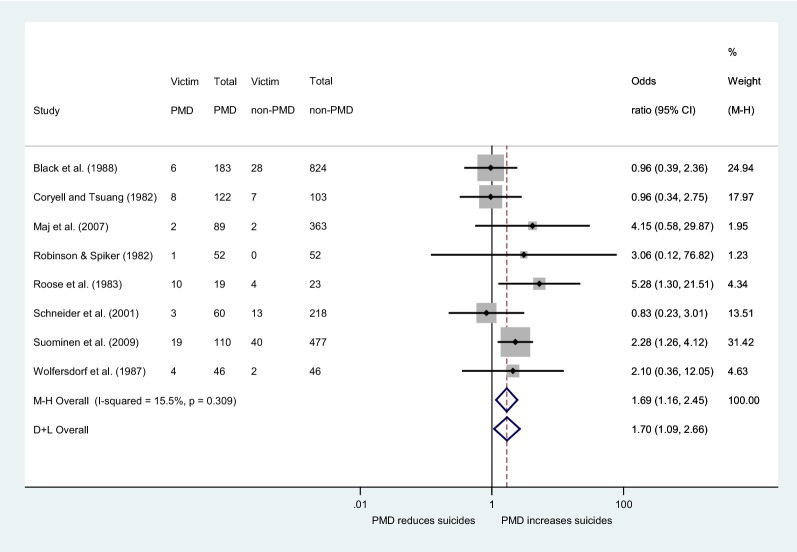
Forest plot of study-specific odds ratios for completed suicide excluding the study of Leadholm et al. [17]. The term ‘M–H’ stands for the fixed-effect approach using the Mantel–Haenszel method, whereas the term ‘D + L’ stands for the random-effects approach

As the fixed-effect pooled estimate was dominated by the Leadholm et al.’s [17] large study which accounts for the 86.62% of the total weight (Fig. 2), we performed a sensitivity analysis excluding this study. Results showed that the statistically significant difference between the two groups persisted and it became even stronger: the fixed-effect pooled OR was 1.69 (95% CI 1.16–2.45), with the *I*^2^ statistic being 15.5% indicating weak (and non-statistically significant *p* = 0.309) heterogeneity, whereas the random-effects pooled OR was 1.70 (95% CI 1.09–2.66) (Fig. 3). In the main analysis, there was no significant small-study effect as evaluated by the Egger’s test (*p* = 0.782) and the corresponding funnel plot.

### Results from the systematic review leg of the study

#### Suicides in relation to patients’ age

The included studies evaluated suicides in the range of adulthood. Three studies [11, 14, 63] did not provide specific data regarding the age of PMD or non-PMD patients, whereas the remaining six studies [12, 16, 17, 19, 62, 63] reported mean ages of their samples within the range of adulthood (Table 1). No study was found with data on suicide in children or adolescents. Given that the included studies provide data on suicidality within the age of young adulthood, it was not possible to study suicides among the elderly people as a separate category.

**Table 1:** Characteristics of included studies

Authors	Study type	Study source/country	Age PMD/non-PMD	Sample: PMD/non-PMD	Suicide victims PMD/non-PMD	Strengths	Limitations	Suicides in PMD vs. non-PMD
Black et al. [14]	Clinical, 0–14-year prospective/retrospective	Initially inpatients, Iowa, USA	No data	183/824	6/28	Adequate initial assessment of psychosis	Lack of data on: diagnosis at the time of death, depression severity, comorbidity, mood congruence, suicide method	No difference
Coryell and Tsuang [16]	Clinical, 40-year, prospective/retrospective	Initially inpatients Iowa, USA	43.8/43.9	122/103	8/7	Adequate initial assessment of psychosis	Lack of data on: diagnosis at the time of death, depression severity, comorbidity, mood congruence, suicide method	No difference
Leadholm et al. [17]	Population-based, nation-wide (in Denmark), prospective/retrospective 17-year follow-up	Patients treated at hospital-based settings Denmark	No specific data (age at diagnosis of severe depression all samples 51.2 (SD 18.9)	12,150/26,106	280/551	Large study comprising all patients discharged from hospital-based settings during 17 years in Denmark, data on comorbidity, mood congruence, and severity	ICD-10 criteria, lack of psychiatric diagnosis at the time of death and on suicide method	No difference
Maj et al. [19]	Clinical, 10-year follow-up	Patients initially treated at hospital-based setting Naples, Italy	45.2/44.5	89/363	2/2	DSM-IV criteria, assessment both initial at the follow-up period, assessment of severity, mood congruence, and comorbidity	Very small numbers of victims, lack of data on suicide method	Data inconclusive
Robinson and Spiker [62]	Clinical, 1-year follow-up	Initially inpatients, New York, USA	45.6/45.8	52/52 Matched	1/0	Adequate assessment of psychosis	Very small number of victims, no data on severity, comorbidity, mood congruence, suicide method	Data inconclusive
Roose et al. [11]	Clinical, cross-sectional, retrospective	inpatients New York, USA	No data	19/23	10/4	Diagnosis of PMD both initial and at the time of suicide, depression severity	Lack of data on mood congruence, comorbidity, method	PMD ↑ suicides
Schneider et al. [64]	Clinical, 5-year follow-up	Initially inpatients, Frankfurt Germany	No specific data, mean of the sample 39.8 (SD 16.8) range 11–74	60/218	3/13	DSM-III-R criteria and data on severity, psychiatric comorbidity, and mood congruence	Lack of psychiatric diagnosis at the time of death, no data on suicide method, small number of victims	No difference
Suominen et al. [12]	Clinical, prospective/retrospective, 4-year follow-up	Initially inpatients with suicidal attempt, Helsinki, Finland	No specific data (mean of the sample 41.7 SD 15.4) Range ≥ 16 years old	110/477	19/40	Assessment of depression severity	ICD-10 criteria, lack of psychiatric diagnosis at the time of death. No data on comorbidity, mood congruence and suicidal method	PMD ↑ suicides
Wolfersdorf et al. [63]	Clinical, cross-sectional, retrospective	inpatients, Ulm, Germany	No data	46/46	4/2	Initial assessment of psychosis and assessment at the time of suicide	Lack of suicide definition, and data on comorbidity	Data inconclusive

#### Suicides in relation to the phase of the disorder, acute, or in remission

The study of Roose et al. [11] has shown an increased risk for suicide in hospitalized or recently discharged PMD patients (10 victims out of 19 patients) compared to non-PMD (4 victims out of 23 patients) with an estimated OR = 5.3. Wolfersdorf et al. [63] following the same methodology with the Roose et al.’s [11] study found that four PMD and two non-PMD patients of their sample committed suicide, numbers too small any conclusion to be drawn. All the remaining studies were follow-up studies which have assessed the overall risk for suicide of PMD as compared to non-PMD patients in short [62] or long term [12, 14, 16, 17, 19, 64]. Thus, these studies did not display data on suicide risk separately for the acute phase of the disorder or in remission.

#### Suicides in relation to method used (violent or not) and mood congruence

The studies included in the meta-analysis do not provide data on the suicide method used, and only the psychological autopsy study of Isometsa et al. [61]—which was included in the systematic review leg of the study—reported that, in all suicides committed within 1 year in Finland, PMD patients were more likely to commit more violent suicides than non-PMD patients (21 cases out of 26 PMDs [88%] vs. 27 cases out of 46 [59%]). Violent suicide methods included hanging, shooting, and cutting, whereas nonviolent included drowning, use of gases or intoxicants, drugs, and poisons.

As regards the contribution of mood congruence in suicide risk, Kessing [60] found no differences in the risk of suicide between PMD patients with or without mood incongruent psychotic features. In the Maj et al.’s [19] study, the small number of suicide victims (two PMD patients and two non-PMD) did not allow any meaningful comparisons between the 58 PMD patients with mood congruent psychotic features and the 21 with mood incongruent ones. In the Schneider et al.’s [64] study, 2 out of 52 PMD patients with mood congruent psychotic features committed suicide and 1 out of 8 PMD patients with mood incongruent ones. As it is obvious, the small numbers of patients in these studies do not allow meaningful comparisons.

#### Suicides in relation to comorbidity

In the Leadholm et al.’s [17] study, it was found in both PMD and non-PMD patients that the odds of committing suicide were lower if the suffered from an organic mental disorder, albeit this relation was stronger in PMD. In addition, Isometsa et al. [61] in a psychological autopsy study did not find that that the two groups differed in regard to anxiety, psychoactive substance, physical illness, and personality disorder comorbidity.

## Discussion

This systematic review and meta-analysis of nine studies, comprising in total 33,873 patients, among them 828 suicide victims, showed that patients with PMD are at 1.21-fold higher lifetime risk of committing suicide than patients with non-PMD. An additional sub-analysis of eight studies comprising 2787 patients, among them 149 suicide victims, after the exclusion of a large study with a huge weight in the analysis (86.62%) revealed a 1.69-fold increased risk for suicide of PMD patients, as well.

Our findings have important clinical implications. Patients with PMD during a major depressive episode experience psychotic phenomena, impulsivity, and strong feelings of guilt, shame, intense anxiety, fear, etc., being in a chaotic mental state and unable to control their actions and suicidal behavior [53]. Unfortunately, these phenomena and symptoms are frequently underdiagnosed as these patients are often too psychomotorly disturbed and avoid giving adequate verbal information about their psychotic experiences, symptoms, and suicidal thoughts [65], and thus, their suicidal intention remains frequently overlooked. On the other hand, our study reports alarming findings on suicide risk of PMD patients and highlights the importance of recognizing and treating adequately psychotic depression and assessing patients’ suicidality, as well [66].

To the best of our knowledge, only the review of Zalpuri and Rothschild [21], comprising eight studies [11, 12, 14, 16, 17, 61–63] has investigated the effect of psychosis on suicide risk using a systematic research method without a meta-analytic leg though. The authors conclude that “most studies did not find any differences in completed suicides between the two groups; however, in some studies, the psychiatric status of the victims at the time of suicide was unknown”. The systematic part of our study comprised 12 studies (four more studies: 13, 19, 60, 64), while the meta-analytic part, which quantified the effect of psychosis on unipolar depressive patients suicide behavior, included nine studies [11, 12, 14, 16, 17, 19, 62–64]. We also attempted to investigate the associations between PMD’s suicides with age, severity of depression, phase of the disorder, mood congruence, suicidal method used, and comorbidity.

The overall results of our main meta-analysis of nine studies showed an increased 1.21-fold lifetime risk of suicide for PMD patients compared to non-PMD. We consider this finding as very interesting: so far, the results of large and influential studies did not show any differences between the two groups of patients in the long term [14, 16, 17], and thus, the above-mentioned comment of Zalpuri and Rothschild [21] seemed to be justified. Furthermore, the finding of our main meta-analysis attempts to answer whether psychosis is a risk factor for suicide in the context of unipolar depression, a question which is often encountered in the literature. We consider this research question as quite general in nature. In the current meta-analysis, two different sorts of studies were included: two studies [11, 63] investigated the suicide risk of PMD patients during the acute phase of the disorder and the remaining seven studies presented the long-term suicide risk of PMD patients. In the Roose et al.’s [11] study, PMD patients were found to be 5.3 times as likely to commit suicide compared to non-PMD patients. The patients of this study committed suicide while hospitalized in the inpatient setting or after having recently eloped or while out on pass. Therefore, they were thoroughly assessed for their psychopathological status at the time of suicide. In addition, Wolfersdorf et al. [63] following the same methodology with the above-mentioned study found only very small and inconclusive numbers of suicide victims. The results of Roose et al.’s [11] study are in line with the findings of our recently published meta-analysis of 20 studies [20] which found a twofold increase of suicide attempt during the acute phase. The authors of the Roose et al.’s [11] study attempt to answer the question “is the risk for suicide of PMD compared to non-PMD patients increased during a major depressive episode?” We consider this question as the most crucial in clinical practice and their results are alarming at the highest degree. Of note, the weight of the Roose et al.’s [11] study in the meta-analysis was extremely low (0.58%) and Wolfersdorf et al. [63] 0.62%, as well, and thus, practically, the pooled OR of our main analysis is extracted from the remaining seven studies. Surprisingly, the results of the Roose et al. [11] have not been replicated in a large study yet, and this certainly constitutes a major weak point in PMD’s suicidal behavior research.

The remaining seven studies reported data on short- [62] or long-term time period [12, 14, 16, 17, 19, 64]. These studies assessed initially patients as either PMD or non-PMD, without providing information over the presence of psychosis in patients’ depressive episodes during the course of the disorder, with the exception of the Leadholm et al.’s [17] study which was a registered-based nation-wide study and followed patients within a period of 17 years providing data over the number of patients with psychotic or non-psychotic episodes or with both. These studies followed up their patients without providing separate risk values for suicide for the acute phase of the disorder or the remission though, attempting thus to answer the question “is the risk for suicide of PMD compared to non-PMD patients increased in the long-term?”. Given the much lower fixed-effect pooled OR of our main meta-analysis in comparison to the Roose et al. [11] study, there is an “attenuation” in the risk of suicide between the study of the acute phase [11] to the studies offering lifetime data.

In an attempt to interpret this previously mentioned difference, we should take into consideration several parameters: these studies consisted of initially hospitalized patients, gathering information about suicides from national data bases [14, 17], or by interviewing living probands and first-degree relatives even many years after death [16]. In these studies, retrospective in nature, the patients’ psychopathological state at the time of suicide remains unknown, which constitutes a major limitation. Even studies which closely followed initially admitted patient in short- [62], or long-term [19] follow-up do not provide an accurate diagnosis at the time of suicide. In addition, patients on long-term follow-up receive antidepressants and for long periods of time are in remission.

Furthermore, the diagnoses of PMD and/or non-PMD are not stable: in the Kessing [60] study, only 50% of patients with a single PMD episode re-presented the same symptoms in their second admission, and in the Ruggero et al.’s [50] study, PMD patients shifted to other diagnoses at a rate over 40% across 10 years. Coryell and Tsuang [16] determined a switch to bipolarity at a rate of about 12.5% for both PMD and non-PMD patients and Maj et al. [19] at 10.1%. Moreover, we should take into consideration that, in the course of the disorder, patients present not only PMD but also non-PMD episodes [67]. Finally, the Leadholm et al. [17] and the Suominen et al. [12] studies used ICD-10 criteria for PMD that include beyond delusions or hallucinations depressive stupor, as well.

It should also be underlined that Schatzberg and Rothschild [68] presented data in favor of poorer short-term outcome (< 2 years) of PMD patients compared to non-PMD patients; however, their long-term outcome (> 2–5 years) was found to be similar to their non-PMD counterparts. Studies that compared the course and outcome of PMD to non-PMD patients have found PMD patients to present more enduring depressive episodes, more depressive episodes, fewer weeks in remission, higher frequency of relapse of recurrence, higher number of hospitalizations, more psychological impairment, higher rates of alcoholism, and a twofold greater risk of death [see, for review, 8, 68, 69]. However, other studies found similar rates between the two groups with regard to the number of depressive episodes and hospitalizations [70] and global functioning at the end of the follow-up period [19, 71]. We consider that the suicide risk in PMD follows a similar pattern to the impact of psychosis in the short- or long-term course of the disorder as presented by Schatzberg and Rothschild [68]. Consequently, these follow-up studies might present an attenuation in PMD’s suicide OR due to the long periods of patients in remission, use of antidepressants, lack of accurate diagnosis at the time of suicide, PMD’s diagnostic instability, and the use of ICD-10 criteria.

At this point, we should comment that the Leadholm et al.’s [17] study is by far the largest study included in the meta-analysis, carried out in Denmark, following up the course of 34,671 PMD and non-PMD patients, with 755 of them committing suicide, without finding any statistically significant differences between the two groups. Nevertheless, the inclusion of this interesting study raises two methodological questions. First, its weight in the meta-analysis is huge (86.62%), and thus, its overall results tend to determine the findings of the meta-analysis, at a crucial degree. Furthermore, the PMD and non-PMD diagnoses were not mutually exclusive, leading thus to the inclusion of 3585 patients in both PMD and non-PMD groups, among them 76 who committed suicide. To adjust for any effects due to patients appearing in both groups, in our main meta-analysis, we removed the numbers of patients with both diagnoses. We speculate that this removal was necessary not only to avoid the inclusion of 3585 patients in both groups, but also to reduce the probability of the type II error. More specifically, the ICD-10 criteria for psychosis include depressive stupor and tend either way to categorize, in the PMD group, a number of patients with no real psychosis, but with only severe psychomotor retardation. When in the PMD group we included patients with both ICD-10 PMD and non-PMD episodes, this tendency to type II error might have become even stronger. We consider that, for this reason, our main meta-analysis showed both the fixed and random-effects being statistically significant, whereas, in the last one, the fixed-effect models remained statistically significant, but the random-effects were marginally not significant.

Owning to the previously mentioned considerations, we performed a sub-analysis excluding the above-mentioned large study and including the remaining eight studies. PMD patients were found to be 1.69 times more likely to commit suicide compared to non-PMD ones. This sensitivity analysis comprised studies of four countries (USA, Germany, Italy, and Finland) with even more alarming results. The strong point of this sensitivity analysis lies on the low heterogeneity of the included studies with respect to research question. On the other hand, the total number of included patients was reduced, and this constitutes its weak point.

Furthermore, it should be noted that the three excluded studies, namely the Isometsä et al. [61] psychological autopsy study and the population-based Brådvik et al. [13] study presented data which showed a preponderance of PMD patients among suicide victims. The third study, in particular the Kessing [60] one, did not report statistically significant findings, but its sample was part of the Leadholm et al. [17] study.

### Suicides in association with age and depression severity

Our search did not isolate any studies concerning PMD’s suicides in adolescence and in old age. Only Schneider et al. [64] and Suominen et al. [12] included some patients over 11 or 16 years, respectively, and an undetermined number of elderly people, as well. In general, we consider that all studies included patients within the age range of adulthood.

The investigation of the contribution of depression severity to PMD patients’ suicides was between the purposes of our study. At this point, it should be noted that our main analysis is conducted between patients suffering from severe depression. Leadholm et al. [17] and Suominen et al. [12] compared both groups of patients suffering from severe depression and the weight of these two studies in the main meta-analysis is over 90%. As, we analytically mention in our Results, the clinical characteristics of Black et al. [14], Coryell and Tsuang [16] and Schneider et al. [64] studies, which do not offer data on depression severity and occupy an additional 7.55% weight in the main meta-analysis, imply the inclusion of patients suffering to a great extent of severe depression, as well. The remaining studies share the same clinical characteristics but offer only a small number of individuals in the analysis. In other words, our main meta-analysis may show that the existence of psychosis in the context of severe depression elevates the risk of suicide. In addition, the Suominen et al.’s [12] study displayed data on suicides across different degrees of depression severity, and as we calculated in the Results, this study clearly showed that the greater the depression severity, the higher the risk for suicide. Moreover, it showed that psychosis in the context of severe depression doubles the risk of suicide. However, not all studies are in agreement: Kessing [60] comparing PMD patients to all non-PMD patients (ICD-10 criteria), regardless of depression severity, did not find any statistical significant differences in terms of suicides. Thus, our meta-analysis reports that PMD patients manifest elevated risk of suicide even when they are compared with non-PMD patients who suffer from severe depression. However, the ICD-10 categorization of PMD only in the context of severe depression and the inclusion of depressive stupor in the criteria of psychosis constitute obstacles on the further elucidation of suicide risk, PMD and depression severity association.

### Suicide and method used (violent or not), mood congruence, and comorbidity

The systematic review leg of this study revealed a psychological autopsy study [61] of all suicides committed within 1 year in Finland, which found that PMD patients were more likely to commit violent suicides than non-PMD patients. In the Suominen et al.’s [12] study, patients with severe depression used a more violent method; however, the authors do not provide data separately on the suicides committed by PMD and/or non-PMD patients. As regards suicidal attempts, Hori et al. [35] have reported significantly higher levels of violent suicidal attempts in PMD patients, and Coryell et al. [15] found that “non-PMD patients were twice as likely to have made attempts judged to be medically and psychologically non- serious”. In the same vein, Lyness et al. [38] have shown that patients with PMD committed more “severe” suicidal attempts in comparison to non-PMD patients. On the other hand, in a study of our group [18], no differences were found between the two groups in terms of the method used. Thus, more research is warranted to clarify the effect PMD exerts on suicide method used.

In respect to the mood congruence of psychosis on suicide risk, Kessing [60] found no differences in the risk of suicide between PMD patients with or without mood congruent psychotic features. In the Maj et al. [19] and in the Schneider et al. [64] studies, the numbers of PMD patients with or without mood congruent psychosis who committed suicide are too small to be conclusive. In addition, our recent meta-analysis [20] on suicidal attempts found the evidence scarce on this topic. Moreover, rather the high prevalence of mixed psychotic features in PMD, 58% in the Burch et al.’s [72] study underlines the restrictions that research work can face to clarify the differential effect of mood congruent vs. mood incongruent psychosis on suicide risk evaluating the two concepts in a categorical way.

Surprisingly, only two studies [17, 61] assessed a number of parameters regarding psychiatric or physical comorbidity such as anxiety, psychoactive substance use, physical illness, or personality disorder but found only limited associations: in the Leadholm et al.’s [17] study, PMD patients were less likely to committing suicide if they suffered from an organic mental disorder. Consequently, the evidence at this point is restricted to the findings of the afore-mentioned two studies, and thus, no conclusions on this topic can be drawn [73].

### Limitations

In addition to the methodological issues discussed above regarding the studies included, such as lack of accurate diagnosis at the time of suicide, PMD’s diagnostic instability, and the use of ICD-10 criteria, further limitations of this study are the search of relevant articles only in English literature and the inclusion of studies carried out in different time periods with possibly different general trends in suicidal behavior. On the other hand, we performed a large search in English literature and in the so-called “gray literature” as well, including, in our meta-analysis, 9 studies with a large number of patients. Furthermore, we consider the clarification of the research questions as a crucial contribution to the field.

## Conclusions

The present systematic review and meta-analysis has shown that psychosis in unipolar depression is an important risk factor for suicide. This risk is elevated in both the acute phase of the disorder and lifetime. Consequently, PMD should be early and accurately diagnosed, and a possible concurrent suicidal ideation thoroughly evaluated. Then, effective treatment for the disorder and suicide management plans should be implemented. Future studies should assess the diagnosis of patients close to the time of suicide more accurately, should evaluate the impact of psychosis (delusions and/or hallucinations) on suicide in adolescence and old age, and explore further the role of depression severity, and the role of psychiatric, substance, and physical comorbidity in PMD’s suicide behavior, as well.

## Additional file

**Additional file 1.** Additional figures.

## Abbreviations

PMD: (unipolar) psychotic major depression
HPA: hypothalamic–pituitary–adrenal (axis)
OR: odds ratio
