# Supplementary material for: Psychotic (delusional) depression and completed suicide: a systematic review and meta-analysis
Source: Ann Gen Psychiatry. 2018 Sep 21;17:39. doi: 10.1186/s12991-018-0207-1 (PMC6150953; doi:10.1186/s12991-018-0207-1)
Supplement: Supplementary file 1 — Additional file 1. Additional figures. [file 12991_2018_207_MOESM1_ESM.doc]

**Gournellis et al. Psychotic (Delusional) Depression and Completed Suicide: a Systematic Review and Meta-Analysis**

**Additional material**

Figure S1. Funnel plot of study-specific odds ratios. The vertical axis contains a measure of precision associated with each study estimate and the horizontal axis contains the odds ratio on the log scale. Thus, larger studies are generally higher on the vertical axis (main analysis, corresponds to Figure 2 of the paper)

Egger’s test p-value = 0.141

Figure S2. Funnel plot of study-specific odds ratios excluding the study of Leadholm et al. (2014). The vertical axis contains a measure of precision associated with each study estimate and the horizontal axis contains the odds ratio on the log scale. Thus, larger studies are generally higher on the vertical axis (sensitivity analysis, corresponds to Figure 3 of the paper).

**Forest and funnel plots including patients’ numbers as presented in Leadholm et al [17] study and all patients of Suominen et al [12] study regardless disease severity**

Figure S3. Forest plot of study-specific odds ratios for completed suicide irrespective of disease severity. The term 'M-H' stands for the fixed-effects approach using the Mantel-Haenzel method, whereas the term 'D+L' stands for the random effects approach.

Fixed-effects p-value = 0.020

Random-effects p-value = 0.049

Figure S4. Funnel plot of study-specific odds ratios irrespective of disease severity (all patients of Leadholm et al. [17] and Suominen et al [12] studies). The vertical axis contains a measure of precision associated with each study estimate and the horizontal axis contains the odds ratio on the log scale. Thus, larger studies are generally higher on the vertical axis.

Egger’s p = 0.160

**Forest and funnel plots including patients’ numbers as presented by Leadholm et al. [17] study and the Suominen et al [12] patients with severe depression)**

Figure S5. Forest plot of study-specific odds ratios for completed suicide in patients with severe depression. The term 'M-H' stands for the fixed-effects approach using the Mantel-Haenzel method, whereas the term 'D+L' stands for the random effects approach.

Fixed-effects p-value = 0.036

Random-effects p-value = 0.057

Figure S6. Funnel plot of study-specific odds ratios in patients with severe depression. The vertical axis contains a measure of precision associated with each study estimate and the horizontal axis contains the odds ratio on the log scale. Thus, larger studies are generally higher on the vertical axis.

Egger’s p = 0.112

**Forest and funnel plots including PMD patients of Leadholm et al.[17] study with only PMD episodes and both PMD and non-PMD episodes compared to patients with only non-PMD episodes and the Suominen et al. [12] patients with severe depression**

Figure S7. Forest plot of study-specific odds ratios for completed suicide. The term 'M-H' stands for the fixed-effects approach using the Mantel-Haenzel method, whereas the term 'D+L' stands for the random effects approach.

**Fixed-effects p-value = 0.037**

**Random-effects p-value = 0.057**

Figure S8. Funnel plot of study-specific odds ratios. The vertical axis contains a measure of precision associated with each study estimate and the horizontal axis contains the odds ratio on the log scale. Thus, larger studies are generally higher on the vertical axis.

**Egger’s p = 0.114**

**Appendix**

**Search Strategy**

We performed a systematic review of the literature using the following search terms to search for all relative studies in Pubmed: suicid* AND ((psychotic depression) OR (delusional depression) OR (depression with psychotic features) OR (depression with psychotic*)); and in Scopus: (TITLE-ABS-KEY (suicid*)) AND ((TITLE-ABS-KEY (psychotic depression) ) OR (TITLE-ABS-KEY (delusional depression)) OR (TITLE-ABS-KEY (depression with psychotic features)) OR (TITLE-ABS-KEY (depression with psychotic*))) respectively.
